# Anti-Mycobacterial Antibiotic Therapy Induces Remission in Active Paediatric Crohn’s Disease

**DOI:** 10.3390/microorganisms8081112

**Published:** 2020-07-24

**Authors:** Gaurav Agrawal, Harrison Hamblin, Annabel Clancy, Thomas Borody

**Affiliations:** Research Department, Centre for Digestive Diseases, Five Dock, NSW 2046, Australia; Harrison.Hamblin@cdd.com.au (H.H.); Annabel.Clancy@cdd.com.au (A.C.); Thomas.Borody@cdd.com.au (T.B.)

**Keywords:** Crohn’s disease, paediatric, Mycobacterium avium paratuberculosis, Antibiotic Therapy

## Abstract

Crohn’s disease is increasing in incidence and prevalence in younger people and is of a particularly aggressive nature. One emerging treatment targets *Mycobacterium avium paratuberculosis* (MAP), an organism implicated in the causation of Crohn’s disease. This study reviewed a cohort of paediatric patients with active Crohn’s disease treated with Anti-Mycobacterial Antibiotic Therapy (AMAT). Sixteen paediatric patients, the majority of whom had failed conventional immunosuppressive therapy, were treated with AMAT. Endoscopic remission was scored using the Simple Endoscopic Score for Crohn’s Disease and clinical remission was assessed using the Weighted Paediatric Crohn’s Disease Activity Index (wPCDAI). Inflammatory blood markers were also routinely recorded. Patients were followed up clinically and endoscopically during treatment after an average of two months (range 1–6) and 17 months (range 2–49), respectively. A significant reduction in both scores assessing clinical improvement (*p* < 0.001) and mucosal healing (*p* < 0.0078) was observed at these timepoints; 47% of patients had achieved clinical remission and 63% endoscopic remission. Haemoglobin and serum inflammatory markers normalised for more than 50% of the cohort by six months of treatment. No adverse effects were reported throughout treatment. This is the first report of Anti-Mycobacterial Antibiotic Therapy offering a safe and efficacious therapy for paediatric patients with Crohn’s disease. Further larger randomised studies are required in order to validate these findings.

## 1. Introduction

Crohn’s disease (CD) is an incurable chronic inflammatory disease occurring predominantly in the gastrointestinal tract and is characterized by deep ulceration, skip lesions, transmural inflammation, fistulae and non-caseating granulomas [1]. CD has a damaging effect on many aspects of a patient’s quality of life and has been shown to be more pronounced in paediatric patients [2,3]. Recent research suggests that newly industrialised countries account for the majority of the increase in paediatric CD incidence on a global scale [4].

Exclusive enteral nutrition (EEN) is recommended as the first line therapy for paediatric CD, with mucosal healing rates ranging from 19% to 75% [5]. Despite this, the long-term efficacy and applicability of EEN is limited with a reported relapse rate of 58% [6]. Oral corticosteroids are used for the induction of remission in moderate to severe paediatric CD cases [5]. However, their use is limited due to their inability to promote significant levels of mucosal healing in patients [7,8] and can lead to serious adverse events [5,9]. Anti-TNF (tumor necrosis factor) agents, as part of a treatment group known as biologics, can achieve mucosal healing both to a higher degree and more rapidly [10]. However, there are concerns regarding the safety and long-term effectiveness of biologics. A loss of response, mediated by the formation of antibodies, occurs in up to 36% of patients within one year of starting treatment [11]. Other notable risks associated are psoriasis, with an incidence of 16% and opportunistic infections such as sepsis, meningitis and pneumonia which have been reported in 3.3% of paediatric patients [5,12].

The sudden emergence of CD in traditionally low-prevalence regions such as Asia suggests that the development of CD may be influenced by environmental determinants [13]. One specific bacterium, *Mycobacterium avium* subspecies *paratuberculosis* (MAP), has been suggested to be a prominent environmental factor in the pathogenesis of CD [14]. Indeed, several studies have observed strong correlations between the incidence of Johne’s disease; an inflammatory granulomatous enteritis of livestock known to be caused by MAP and the rates of CD in the same geographic region [15,16,17]. These studies formed vital pieces of evidence employed by Chiodini in 2012 to describe MAP’s fulfilment of Bradford Hill’s Criteria for Causation in CD [18,19].

Recent increasing evidence about the involvement of MAP in the pathogenesis of CD has led to the development of a targeted and specialized antibiotic regimen termed Anti-Mycobacterial Antibiotic Therapy (AMAT). AMAT consists of four antibiotics, with rifabutin, clarithromycin and clofazimine the mainstay; and nitroimidazole or quinolone making up the fourth depending on the clinical presentation. Large randomized controlled studies assessing this precise antibiotic regimen were limited until a recent phase-three trial, where at week 26, 37% of patients who received standard therapy with added AMAT entered clinical remission compared to 23% in the control group who received standard therapy with the addition of a placebo [20].

The rapid rise in CD prevalence and the lack of safe and effective treatment options for children with CD prompts the need for an alternative, yet effective treatment modality. Recent evidence surrounding the pathogenic role of MAP in CD and the effectiveness of AMAT in the adult population with CD warrants further investigation into its applicability to the paediatric cohort. This study aims to examine both the safety and efficacy of AMAT in paediatric patients with active CD.

## 2. Materials and Methods

A retrospective review of all paediatric patients with CD at our centre seen between the period of January 2004 and March 2018 was conducted in February 2019. Clinical data were collated from medical records including demographics (gender and age at AMAT commencement), previous treatment for CD, AMAT regimen and any adverse events. Immunosuppressive medications including anti-TNF, steroidal, immunomodulatory and 5-aminosalicylic acid (5-ASA) that were prescribed concomitantly were also recorded (Supplementary Table S1). Patients received an escalating dosing regimen to average maximum doses of rifabutin 10.9 ± 0.7 mg/kg/d, clofazimine 2.4 ± 0.2 mg/kg/d and clarithromycin 26.4 ± 2.1 mg/kg/d (Table 1). This triple therapy was combined with up to two other antibiotics, most commonly, metronidazole 8.7 ± 0.6 mg/kg/d or ciprofloxacin 32.5 ± 4.6 mg/kg/d (Table 1).

Due to its reported superiority in retrospective analysis and greater correlation with measures of endoscopic inflammation when compared to alternative scores, [21,22] the Weighted Paediatric Crohn’s Disease Activity Index (wPCDAI) score was used to calculate patient clinical improvement while on AMAT. The overall score classifies patients into four disease activity categories: clinical remission, <12.5; mild, 12.5 to 40; moderate, >40 to 57.5; and severe, >57.5 [23]. Scores were calculated prior to treatment commencing and at every follow up appointment thereafter within the first year of treatment. The primary outcome was defined as clinical remission at the first follow-up appointment following the commencement of AMAT. Patients whose clinical data did not satisfy all of the parameters required for the wPCDAI score were excluded from scoring and subsequent analysis.

The Simple Endoscopic Score for Crohn’s Disease (SES-CD) was deemed the most appropriate tool for evaluating patient endoscopic response [24,25]. In this study, a SES-CD score between 0 and 2 suggested endoscopic remission, 3 to 6 mildly active, 7 to 15 moderately active and ≥16 severely active disease [25]. SES-CD scores were calculated prior to and throughout treatment using photographs taken at the time of the colonoscopy. The primary outcome was endoscopic remission at the first colonoscopy after the commencement of AMAT. Patients whose colonoscopy reports did not include supporting photographs of all segments of the large bowel were excluded from scoring and subsequent analysis.

Serum C-reactive protein (CRP), erythrocyte sedimentation rate (ESR), alkaline phosphatase (ALP), aspartate transaminase (AST), alanine transaminase (ALT), hemoglobin (Hb), ferritin, white cell count (WCC), albumin and vitamin D were recorded prior to and at 1, 6 and 12 months into treatment.

Statistical considerations: statistical analysis and graphing was conducted using GraphPad Prism version 8.1.1 for Windows, GraphPad Software, San Diego, California USA, www.graphpad.com. Descriptive statistics were calculated and tabulated. The Wilcoxon signed rank test was used to compare the differences between wPCDAI and SES-CD scores prior to and at the first clinical and colonoscopy follow up appointments. Fisher’s exact test was used to examine the contributory effects of 4 classes of concomitant immunosuppressive medications: anti-TNF, steroidal, immunomodulatory and 5-ASA. Statistical significance was set as *p* < 0.05.

Ethics approval: This study was a retrospective analysis of patients who had sought, consented and were treated with AMAT. The study was submitted to and approved by the institutional ethics committee. A waiver of consent was granted by the Centre for Digestive Diseases Human Research Ethics Committee (CDD19-C01, June 2019) for retrospective review of patient records. Ethics approval for this retrospective analysis was granted by the Centre for Digestive Diseases Human Research Ethics Committee (CDD19-C01, June 2019).

## 3. Results

Sixteen patients (11 male), previously diagnosed with CD were identified to have started AMAT at an average age of 14 years (range: 10 years) (Table 1). The majority of patients (*n* = 14) had been referred to our centre due to failed previous therapies, most commonly immunosuppressive (*n* = 12) (Table 1).

A total of 15 patients for wPCDAI and eight for SES-CD had sufficiently detailed medical records in order to calculate retrospective scores. Prior to AMAT, the median pre-treatment wPCDAI and SES-CD scores were 47.5 (*n* = 15, range: 60) and 24 (*n* = 8, range: 27) respectively (Table 2). There was a statistically significant reduction in wPCDAI scores at the first clinical follow up appointment (*p* < 0.001), where seven (47%) patients achieved clinical remission (Table 2). A further seven patients reached clinical remission at subsequent clinical follow up appointments which occurred after an average of five months since the commencement of AMAT (data not shown). All eight patients scored with the SES-CD displayed a significant reduction when comparing their score prior to treatment and at their first colonoscopy during treatment (*p* = 0.0078). Five of these patients (63%) returned SES-CD scores indicative of endoscopic remission at their first colonoscopy after the commencement of AMAT which occurred after an average of 17 months (Table 2). 

A total of nine (56%) patients were prescribed concomitant medications from a minimum of one of the following immunosuppressive drug classes: anti-TNF, steroidal, immunomodulatory and 5-ASA (Supplementary Table S1). Statistical analysis indicated that concomitant use of either a steroidal and/or immunomodulatory medication during AMAT did not predict clinical and endoscopic remission in such patients. There was an inadequate number of patients prescribed both Anti-TNF and 5-ASA drugs concomitantly in order to perform a Fisher’s exact test to determine a possible synergistic effect.

**Figure 1:**
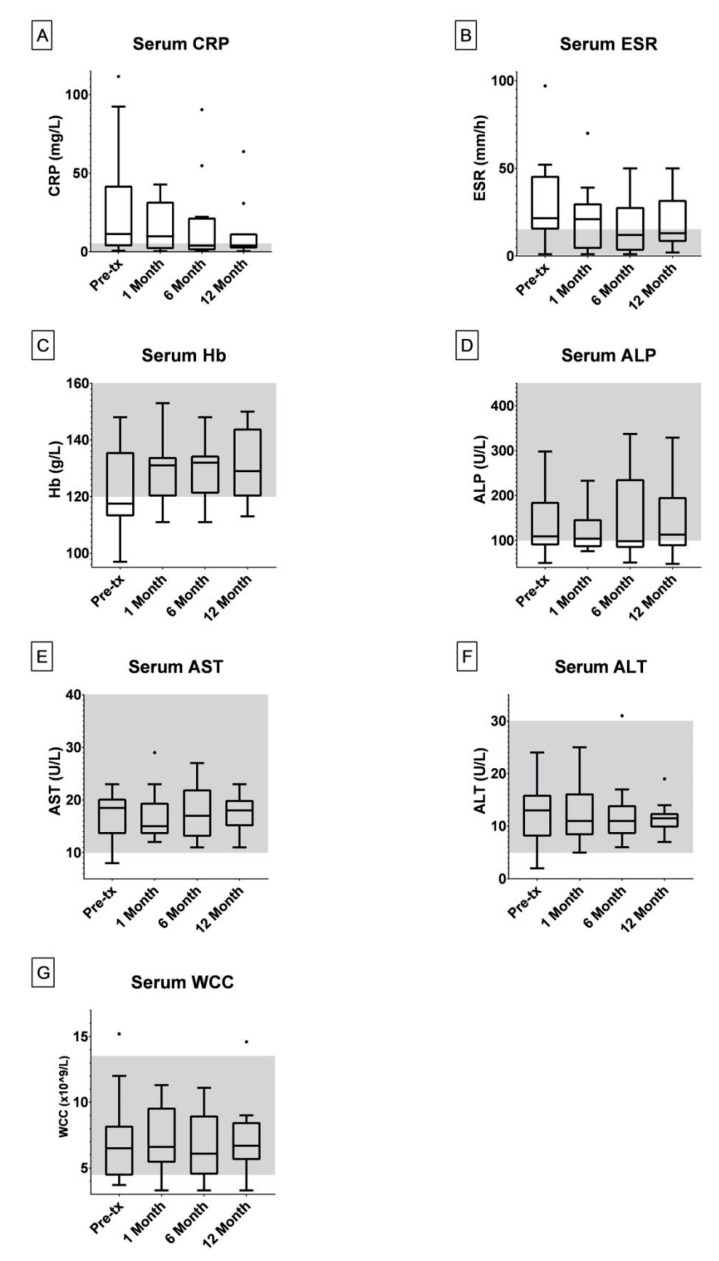
Box and whisker plots were generated in GraphPad Prism 8 under the Tukey method where the median is indicated by the horizontal bar and outliers indicated.‘·’ = The Outlier mark; more than 3/2 times of the upper quartile. The number of patients with blood results for serum C-reactive protein (CRP) (**A**), erythrocyte sedimentation rate (ESR) (**B**), hemoglobin (Hb) (**C**) and white cell count (WCC) (**G**) at pre-treatment, 1, 6 and 12 months during treatment were 16, 15, 13 and 11 respectively. The number of patients with blood results for serum alkaline phosphatase (ALP) (**D**) and alanine transaminase (ALT) (**F**) at pre-treatment, 1, 6 and 12 months during treatment were 15, 14, 12 and 10 respectively. The number of patients with blood results for serum aspartate transaminase (AST) (**E**) at pre-treatment, 1, 6 and 12 months during treatment were 14, 13, 11 and 9 respectively. The standard serum reference ranges for paediatric patients are indicated in grey (CRP: 0–5 mg/L, ESR: 1–15 mm/h, Hb: 120–160 g/L, ALP: 100–450 U/L, AST: 10–40 U/L, ALT: 5–30 U/L, WCC: 4.5–13.5 × 10^9^/L).

The median values for all three of CRP, ESR and Hb serum levels were outside the standard reference range prior to treatment (Figure 1A–C). After six months of AMAT, the median values reduced to within the normal reference ranges for all three serum markers; CRP (0–5 mg/L), ESR (1–15 mm/h) and Hb (120–160 g/L) (Figure 1A–C). Outliers in the CRP and ESR data analysis included patients experiencing secondary infections and non-compliant patients.

Liver function tests (ALP, AST and ALT) as well as WCC showed no significant changes in the population within the first year of treatment (Figure 1D–F). One patient experienced continual fevers while taking rifabutin, however, these were reduced and managed via dosage modulation. Six patients contracted *Clostridioides difficile* infections (CDI) during AMAT, which were treated with fecal microbiota transplantation (FMT).

## 4. Discussion

This retrospective review reports for the first time the safety and efficacy of a combination antibiotic regime targeting MAP in paediatric patients with CD. The results of the present study successfully complement the recent randomised control trial (RCT) assessing the clinical efficacy of AMAT in the adult population [20]. Following our sustained antibiotic regime, we report significant rates of both clinical and endoscopic remission within our paediatric cohort with no significant adverse effects.

Our results in this small cohort indicate that AMAT may be as effective if not more than currently utilised therapies for the induction of clinical and endoscopic remission (Table 2 and Table 3). Although only 47% of patients achieved clinical remission at their first clinical follow-up (Table 2), a total of 93% of patients achieved clinical remission at subsequent follow-up appointments after an average of five months of treatment (data not shown). We report complete mucosal healing in 63% of our cohort at their first follow-up colonoscopy during treatment (Table 2). This result is analogous to the higher rates of endoscopic remission reported in studies using Infliximab though after a longer period of treatment; 17 months compared to a range of 2.5 to 14 months (Table 2 and Table 3, respectively).

There was marked heterogeneity within our cohort specifically surrounding the extent of and type of prior treatment (Table 1). We observed a notable trend whereby patients who had received a greater volume of immunosuppressive therapy prior to AMAT tended to respond both less rapidly and to a lesser extent when compared to those patients who had received fewer or no therapies. Interestingly, immunosuppressives have been shown to have anti-microbial activity, [28,29,30] though non-specific for MAP. Hence, one postulation is that the extent of previous immunosuppressive therapy may predict a longer duration to achieve endoscopic remission under AMAT due to the development of resistant forms of MAP in such patients. Certainly, a recent published report by our group supports this notion, in which eight treatment naïve patients experienced significant rates of both clinical and endoscopic remission at 6 weeks and 12 months respectively [31].

Although a similar combination of antibiotics was used in our patients, a recent phase three RCT assessing the efficacy of AMAT in adult patients utilised a single capsule formulation; RHB-104, which has reported superior efficacy against MAP infections compared with the same individually dissolved drugs [20,32,33]. Despite this, we report greater efficacy in our paediatric cohort compared with the phase three trial in adults; clinical remission rates of 43% at the first clinical follow up (Table 2) and total clinical remission rates of 93% after an average of five months (data not shown) compared with 37% at six months, respectively [20].

The antibiotics utilised within the AMAT protocol reported here are widely used and safe in paediatric patients when the dosage is adjusted for weight. A sun-tanned appearance, nausea and itchiness are the most common side effects reported in patients treated with AMAT constituents, however no such side effects were reported by our cohort. Notable, yet rarer iatrogenic effects, such as leucopenia and elevated transaminases can occur if a dose escalation model is not employed [34,35,36]. However, no significant changes in the WCC and liver function test results (Figure 1D–G) in our patients occurred during AMAT, suggesting that the adopted dose escalation model is suitable. However, larger prospective studies conducted under controlled conditions are required to confirm these results.

A noteworthy finding of our study was the high percentage of patients experiencing CDI whilst being treated with AMAT. However, whether these infections are a result of the antibiotics perturbing the gut microbiome, rendering it susceptible to colonization, remains unclear. It is unlikely that AMAT would encourage the development of CDI since multiple constituents of the therapy; specifically, metronidazole, tinidazole and rifabutin are effective treatments against the infection [37]. Furthermore, paediatric IBD is reportedly associated with a higher incidence of CDI when compared to adults which may partially explain the rates of CDI observed within our cohort [38]. Despite no accounts of increased rates of CDI in previously published studies using AMAT, [20] the authors suggest that future, prospective trials examining the safety of AMAT in the paediatric cohort should take this finding into account.

There are several recognised limitations to this study due to its retrospective design and small sample size. One significant limitation of the present study is that both wPCDAI and SES-CD scores were not calculated at the time of treatment, thus, retrospective calculation may have introduced some level of scoring bias. The lack of a suitable control population for comparison further highlights the uncontrollable limitations associated with retrospective studies and should be employed in future prospective trials. Although no significant synergistic effect was observed in patients who were prescribed concomitant immunosuppressive therapies. Larger, prospective trials should examine the effect these drugs have when used in combination with AMAT compared to AMAT alone.

The present study failed to evaluate the duration of remission in patients due to significant gaps in long-term follow-up data. Therefore, forthcoming RCTs should also focus on establishing the duration of both clinical and endoscopic remission compared to current treatments.

## 5. Conclusions

Despite a well-documented aggressive disease presentation and a recent exponential rise in the incidence of paediatric CD, fewer therapies are available in children when compared to the adult population. We examined the safety and efficacy of a combination antibiotic regime consisting of rifabutin, clofazimine and clarithromycin targeting MAP, a proposed contributing factor in the pathogenesis of CD. This study provides a valuable and novel proof of concept regarding the applicability of AMAT to a broad range of clinical presentations in paediatric Crohn’s disease patients. We report no significant side effects as a result of a dose escalated, sustained antibiotic regimen and suggest that AMAT may be more efficacious in the treatment of childhood onset compared with adult onset CD. Furthermore, we observed a notable trend, which proposes that the extent of prior immunosuppressive therapy may predict a longer treatment course with AMAT. Future targeted and more robust RCTs should also focus on sub-populations in terms of disease severity, extent of prior treatment and the time since initial diagnosis prior to the commencement of AMAT.

## Figures and Tables

**Table 1:** Demographic summary of the paediatric patients treated with Anti-Mycobacterial Antibiotic Therapy (AMAT) (*n* = 16).

Characteristics	Value
**Age and Sex**	
Age at commencement (mean)	14.2
Males (*n*)	11
**Previous Treatment**	
Previous treatment (*n*)	14
Previous immunosuppressive treatment (*n*)	12
**AMAT Drugs (*n*) and Dosage** ($\sigma_{\bar{x}}$ **Max Dose mg/kg/d) used in Combination**	
Rifabutin	16 (10.9 ± 0.7)
Clarithromycin	15 (26.4 ± 2.1)
Clofazimine	16 (2.4 ± 0.2)
Metronidazole	8 (8.7 ± 0.6)
Ciprofloxacin	8 (32.5 ± 4.6)

**Table 2:** Clinical and endoscopic remission rates for the paediatric cohort.

	Patients Achieved at 1st FU *n*	Time to (Months) Mean (Range)	Score Pre-Tx Median (Range)	Score at 1st FU Median (Range)	*p*-Value
**Clinical Remission** (*n* = 15)	7	2 (1–6)	48 (15–75)	18 (0–25)	<0.001
**Endoscopic Remission** (*n* = 8)	5	17 (2–49)	24 (8–34)	0 (0–26)	0.0078

**Table 3:** Comparison of recent reviews examining the effectiveness of current treatment modalities in paediatric Crohn’s disease (CD).

Reference	Studies (*n*) Patients (*n*)	Treatment	Time to Follow Up (Months) Range	Clinical Remission Rates (%) Range	Endoscopic Remission Rates (%) Range
**Kang et al.** (**2018**) [26]	4 (196)	Infliximab	2.5–14	33–89	23–74
**Kang et al.** (**2018**) [26]	2 (31)	Adalimumab	8–14	23	25–42
**Swaminath et al.** (**2017**) [27]	7 (216)	Exclusive Enteral Nutrition	0.5–6	45–87	0–19
**Swaminath et al.** (**2017**) [27]	7 (207)	Corticosteroids	0.5–6	40–100	0

## Data Availability

Data was analysed from the secure practice clinical server and history records. The data that support the findings of this study are available from the Centre for Digestive Diseases, but restrictions apply to the availability of these data, which were used under license for the current study, and so are not publicly available. Data are however available from the authors upon reasonable request and with permission of the Centre for Digestive Disease.

## Supplementary Materials

The following are available online at https://www.mdpi.com/2076-2607/8/8/1112/s1: Table S1: Concomitant Medications Prescribed to Patients During Their Treatment with AMAT.

## Abbreviations

ALP	Alkaline Phosphatase
ALT	Alanine Transaminase
AMAT	Anti-Mycobacterial Antibiotic Therapy
AST	Aspartate Aminotransferase
CD	Crohn’s Disease
CDI	*Clostridioides Difficile* Infection
CRP	C-Reactive Protein
EEN	Exclusive Enteral Nutrition
ESR	Erythrocyte Sedimentation Rate
5-ASA	5-Aminosalicylic Acid
FMT	Fecal Microbiota Transplantation
Hb	Haemoglobin
IBD	Inflammatory Bowel Disease
MAP	*Mycobacterium avium subspecies paratuberculosis*
MTB	*Mycobacterium tuberculosis complex*
RCT	Randomised Controlled Trial
SES-CD	Simple Endoscopic Score for Crohn’s Disease
TNF	Tumor Necrosis Factor
WCC	White Cell Count
wPCDAI	Weighted Paediatric Crohn’s Disease Activity Index
