# Supplementary material for: Anti-Mycobacterial Antibiotic Therapy Induces Remission in Active Paediatric Crohn’s Disease
Source: Microorganisms. 2020 Jul 24;8(8):1112. doi: 10.3390/microorganisms8081112 (PMC7464505; doi:10.3390/microorganisms8081112)
Supplement: Supplementary file 1 [file microorganisms-08-01112-s001.pdf]

Supplementary Table 1: Concomitant Medications Prescribed to Patients During Their Treatment with AMAT

Patient Number	Concomitant Medication	Medication Class	Reason for Prescription
1	Prednisone	Steroidal	To control acute flares
2	None	N/A	N/A
3	Dehydroepiandrosterone	Steroidal	Treatment of only remaining symptom of tiredness
	Sulfasalazine	5-aminosalicylic	Prevention of IDB recurrence after successful initial AMAT
	Mercaptopurine	Immunomodulatory	Weaning from previous treatment
4	Prednisone	Steroidal	To control acute flares
	Azathioprine	Immunomodulatory	Weaning from previous treatment
5	None	N/A	N/A
6	None	N/A	N/A
7	None	N/A	N/A
8	Prednisone	Steroidal	Weaning from previous treatment
	Methotrexate	Immunomodulatory	Weaning from previous treatment
	Budesonide	Steroidal	To control acute flares
9	None	N/A	N/A
10	None	N/A	N/A
11	Prednisone	Steroidal	To control acute flares
12	No Data	N/A	N/A
13	Prednisone	Steroidal	Weaning from previous treatment
	Azathioprine	Immunomodulatory	Weaning from previous treatment
14	Prednisone	Steroidal	Weaning from previous treatment
	Methotrexate	Immunomodulatory	To control acute flares
	Budesonide	Steroidal	To control acute flares
	Mercaptopurine	Immunomodulatory	Weaning from previous treatment
15	Mercaptopurine	Immunomodulatory	To control acute flares
16	Adalimumab	Anti-TNF	Weaning from previous treatment
